# Carcinoma cuniculatum of the foot arising on plantar callus

**DOI:** 10.11604/pamj.2014.18.306.5199

**Published:** 2014-08-16

**Authors:** Mariem Mohamed, Hichem Belhadjali

**Affiliations:** 1 Department of Dermatology, Fattouma Bourguiba University, Hospital of Monastir, Tunisia

**Keywords:** Carcinoma cuniculatum, plantar callus, biops

## Image in medicine

Carcinoma cuniculatum (CC) is a rare subtype of verrucous carcinoma usually affecting the sole of the foot. It was first described in the English-language medical literature by Aird et al in 1954. The incidence of CC worldwide is unknown. It is known to commonly affect males (79-89% of the patients) in their fifties. Carcinoma cuniculatum can arise on chronic ulcer or on recalcitrant plantar wart. The development of CC in keratodermatous skin is a rare event previously reported in severe, extensive areas of keratoderma but it is exceptionally reported in focal plantar keratoderma. It is characterized, clinically, by fungating, exophytic mass with numerous keratin-filled sinuses. We report a case of a man 55 year-old, who was presented at our department with non-healing nodule on the sole. He was working in building sector and he had a long history of plantar callus which caused difficulty in walking. On dermatological examination, we noticed nodular tumor with verrucous surface on the right foot, which measured 3 cm in diameter. The patient had bilateral plantar callus. The diagnosis of carcinoma cuniculatum was then held on the histological examination of a deep biopsy specimen. Magnetic Resonance Imaging (MRI) of the foot showed several focal coalescing soft tissue masses with involvement of flexor tendons fascia and the underlying muscles without bone damage. Consequently, the patient had transmetatarsal amputation. There have been no local recurrences or distant metastasies after a follow-up of 9 years.

**Figure 1:**
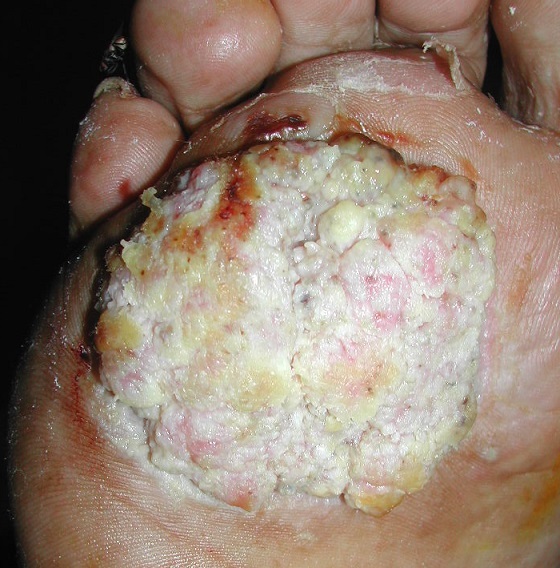
Nodular tumor, 3 cm in diameter with verrucous surface on the right foot

